# Enhanced Heme Function and Mitochondrial Respiration Promote the Progression of Lung Cancer Cells

**DOI:** 10.1371/journal.pone.0063402

**Published:** 2013-05-21

**Authors:** Jagmohan Hooda, Daniela Cadinu, Md Maksudul Alam, Ajit Shah, Thai M. Cao, Laura A. Sullivan, Rolf Brekken, Li Zhang

**Affiliations:** 1 Department of Molecular and Cell Biology, Center for Systems Biology, University of Texas at Dallas, Richardson, Texas, United States of America; 2 Division of Surgical Oncology, Department of Surgery, The Hamon Center for Therapeutic Oncology Research, University of Texas Southwestern Medical Center, Dallas, Texas, United States of America; National Cancer Institute, United States of America

## Abstract

Lung cancer is the leading cause of cancer-related mortality, and about 85% of the cases are non-small-cell lung cancer (NSCLC). Importantly, recent advance in cancer research suggests that altering cancer cell bioenergetics can provide an effective way to target such advanced cancer cells that have acquired mutations in multiple cellular regulators. This study aims to identify bioenergetic alterations in lung cancer cells by directly measuring and comparing key metabolic activities in a pair of cell lines representing normal and NSCLC cells developed from the same patient. We found that the rates of oxygen consumption and heme biosynthesis were intensified in NSCLC cells. Additionally, the NSCLC cells exhibited substantially increased levels in an array of proteins promoting heme synthesis, uptake and function. These proteins include the rate-limiting heme biosynthetic enzyme ALAS, transporter proteins HRG1 and HCP1 that are involved in heme uptake, and various types of oxygen-utilizing hemoproteins such as cytoglobin and cytochromes. Several types of human tumor xenografts also displayed increased levels of such proteins. Furthermore, we found that lowering heme biosynthesis and uptake, like lowering mitochondrial respiration, effectively reduced oxygen consumption, cancer cell proliferation, migration and colony formation. In contrast, lowering heme degradation does not have an effect on lung cancer cells. These results show that increased heme flux and function are a key feature of NSCLC cells. Further, increased generation and supply of heme and oxygen-utilizing hemoproteins in cancer cells will lead to intensified oxygen consumption and cellular energy production by mitochondrial respiration, which would fuel cancer cell proliferation and progression. The results show that inhibiting heme and respiratory function can effectively arrest the progression of lung cancer cells. Hence, understanding heme function can positively impact on research in lung cancer biology and therapeutics.

## Introduction

Tumor cells have an increased demand for nutrients, which provide cellular energy and metabolic building blocks. Increased metabolic demand in tumor cells often accompanies altered metabolism. In the 1920s, Otto Warburg demonstrated that tumor cells metabolize glucose and generate lactate at higher levels despite the presence of ample oxygen, a phenomenon called the Warburg effect [1], [2]. More recent studies have uncovered the molecular events underlying many metabolic alterations in cancer cells [3]–[5]. For example, Anastasiou et al. [5] showed recently that the enzyme pyruvate kinase M2 (PKM2), which is the predominant pyruvate kinase found in cancer cells, is crucial for maintaining cellular redox homeostasis. Furthermore, recent studies suggest that metabolic enzymes can act as tumor suppressors (e.g., fumarate hydratase and succinate dehydrogenase), or oncogenes (e.g., mutant isocitrate dehydrogenase 1 and 2) [6]–[8]. These recent studies confirmed that altered metabolism is indeed a hallmark of cancer, and suggested that the changes in metabolism in cancer cells are much more complex than that was suggested initially.

Recent studies illustrated that enhanced glycolytic flux in cancer cells is not dependent on diminished oxygen consumption or mitochondrial respiration [9]–[11]. For example, two separate studies [12], [13] showed that mitochondrial respiration is amplified in human breast cancer cells. Another study showed that cancer cells can maintain oxidative phosphorylation at a diminished, but still substantial rate even at 1% oxygen level [14]. These results highlight that mitochondrial respiration and function are crucial to cancer cell metabolism and bioenergetics. Particularly, heme is a central factor in mitochondrial function and oxygen metabolism [15], [16]. Heme plays critical roles in virtually every process involved in oxygen metabolism. Heme serves as a prosthetic group in hemoglobin, myoglobin and other globins that transport or store oxygen, in mitochondrial respiratory chain complexes, in cytochrome P450s and other oxygenases that use oxygen for biosynthetic and degradation reactions, and in other enzymes that use or detoxify oxygen such as peroxidases and catalases [15], [17]. Likewise, heme biosynthesis requires oxygen as a substrate, although the Km of heme biosynthetic enzymes for oxygen is very low [18]. Hence, heme and oxygen are tightly linked and interdependent.

Here we investigate the function of heme and mitochondria in lung cancer development. Lung cancer is the leading cause of cancer-related mortality in the US and worldwide, and about 85% of the cases are non-small-cell lung cancer (NSCLC) [19], [20]. Most patients have locally advanced stage III/IV tumors at the time of presentation [21]. Exploitation of metabolic vulnerabilities may provide effective alternative strategies to combat lung cancer progression. We therefore characterized and compared oxygen metabolism and heme function in HBEC30KT and HCC4017 cells [22], [23]. This pair of cell lines represent normal nonmalignant HBEC (HBEC30KT) and NSCLC (HCC4017) cells developed from the same patient. We compared the metabolic and molecular profiles of this matched pair of cell lines grown under identical conditions. We were interested in determining if and to what extent oxygen metabolism and heme levels are altered and if such alterations contribute to the maintenance and proliferation of lung cancer cells. Our data indicate that oxygen consumption and heme synthesis are intensified significantly in lung cancer cells, compared to the normal cells. Additionally, the levels of proteins involved in intracellular heme synthesis and uptake are substantially increased in lung cancer cells. Further, the levels of oxygen-utilizing hemoproteins, such as cytoglobin, were dramatically increased in cancer cells. Inhibition of heme or mitochondrial function preferentially suppressed oxygen consumption, cancer cell proliferation, colony formation and migration. These results show that enhanced synthesis of heme and hemoproteins in lung cancer cells results in intensified oxygen consumption and mitochondrial respiration that fuel cancer cell development.

## Materials and Methods

### Lung Cell Maintenance, Treatment and Cell Count

HBEC30KT and HCC4017 cell lines representing normal and NSCLC cells [22], [23] were provided by Dr. John Minna’s lab (UTSW) as a gift. They were developed from the same patient and were maintained in ACL4 supplemented with 2% FBS under 5% CO_2_ at 37°C [23]. For treatment with the inhibitor of heme synthesis, succinyl acetone, cells were cultured in medium containing heme-depleted serum. Heme depleted serum was prepared as described previously [24]. For measuring the effect of reagents on lung cell proliferation, cells were seeded in 48-well plate at a density of 10^3^ cells/well. After culturing for 24 h, cells were treated with 0.5 mM succinyl acetone or with 10 µM carbonyl cyanide 3-chlorophenylhydrazone (CCCP). Every 24 h post treatment, the number of live cells was counted by using trypan blue staining and a hemocytometer.

### Measurement of Glucose Consumption, Oxygen Consumption and Heme Synthesis

For measuring glucose consumption, cells (∼90% confluence) were incubated for 24 hours with fresh medium. Glucose level in the culture medium was measured using the Glucose (GO) Assay kit (Sigma). Oxygen consumption was measured, as described previously [25]. Briefly, cells with about 80% confluency were trypsinized and resuspended in fresh, air-saturated medium. For each measurement, 10^6^ cells (in 350 µl) were introduced in the chamber of an Oxygraph system (Hansatech Instruments), with a Clark-type electrode placed at the bottom of the respiratory chamber. During measurements, the chamber was thermostated at 37°C by a circulating water bath. An electromagnetic stirrer bar was used to mix the contents of the chamber. Heme synthesis rate was measured as described [24]. Briefly, cells were treated with 0.5 mM succinyl acetone, or 10 µM CCCP for 48 h, and were incubated with 0.3 µCi of [4-^14^C] 5-aminolevulinic acid (PerkinElmer Life and analytical Sciences) for 24 h. Heme was extracted from these cells by using acetone-hydrochloric acid and diethyl ether, and the amount of radiolabeled heme was measured, as described [26]. The incorporation of radioactivity into the extracted heme allows the measurement of heme biosynthesis. The amount of radiolabeled heme was measured by scintillation counting.

### Real-time PCR Quantitation and Detection of Mitochondrial DNA

Oligonucleotide primers for measuring the transcript levels of genes were designed by using the Primer3 program (http://frodo.wi.mit.edu/cgi-bin/primer3/primer3_www.cgi). β-actin was used as a control for the relative quantification of transcripts. Total RNA was extracted from untreated cells or treated with 0.5 mM succinyl acetone in heme-depleted medium by using TRIzol reagent (Invitrogen). RNA was purified by using RNeasy kit (Qiagen). Reverse transcription and PCR were performed in a single step using the LightCycler RNA Master SYBR Green I Kit (Roche Applied Science), according to the manufacture’s protocol. PCR was performed by using a Roche LightCycler. Calculations were done by using the Roche LightCycler software. Primer sequences used for real-time PCR were as follows: β-actin: forward 5′- CACAGGGGAGGTGATAGCAT –3′, reverse 5′- CACGAAGGCTCATCATTCAA –3′. ALAS1: forward 5′-CACACACCCCAGATGATGAA –3′, reverse 5′- CCTGCAGAAGTTGCACTCAG –3′. ALAS2: forward 5′- TGTCACCACCTATGCCTGAG –3′, reverse 5′- GGCACACAACAAAGCAGAAG –3′.

The mtDNA content was measured and compared by quantifying the levels of the mitochondrial 16S rRNA gene and the nuclear GAPDH gene, by using real-time PCR, as described previously [27]. Primer sequences used were as follows (5′-3′): GAPDH: forward TTCAACAGCGACACCCACT, reverse CCAGCCACTACCAGGAAAT. 16S rRNA: forward CCAAACCCACTCCACCTTAC, reverse TCATCTTTCCCTTGCGGTA. Real-time PCR amplification was performed using the LightCycler FastStart DNA Master^PLUS^ SYBR Green I kit (Roche Applied Science), according to the manufacture’s protocol. Real-time PCR amplification for each sample was performed in triplets. Data were collected and analyzed by using the LightCycler Software. The ratio of mitochondrial (16 rRNA) vs. nuclear DNA (GAPDH) was calculated.

### Preparation of Protein Extracts, Western Blotting and Immunofluorescence Microscopy

HBEC30KT and HCC4017 cells were treated, collected, and lysed by using the RIPA buffer (Cell Signaling Technology) containing the protease inhibitor cocktail. Human tumor xenografts were maintained, and lysates from human tumor xenografts were prepared as described [28]. Protein concentrations were determined by using the BCA assay kit (Thermo Scientific). 50 µg proteins from each treatment condition were electrophoresed on 9% SDS–Polyacrylamide gels, and then transferred onto the Immuno-Blot PVDF Membrane (Bio-Rad). The membranes were probed with polyclonal antibodies, followed by detection with a chemiluminescence Western blotting kit (Roche Diagnostics). The signals were detected by using a Carestream image station 4000MM Pro, and quantitation was performed by using the Carestream molecular imaging software version 5.0.5.30 (Carestream Health, Inc.). Immunofluorescence staining was performed by following the procedures provided by the antibody manufacturer. FITC and DAPI fluorescent images were captured by using a multi-channel Zeiss Axio Observer.Z1 fluorescent microscope. Polyclonal anti-ALAS1, anti-HRG1, anti-cytochrome c, anti-cytoglobin, anti-CYP1B1, anti-Cox-2 and anti-HCP1 were purchased from Santa Cruz Biotechnology. Monoclonal anti-β-actin antibody was purchased from Cell Signaling Technology.

### Colony Formation and Cell Migration Assays

For colony formation assay, HCC4017 cells were counted and seeded at a density of 1000 cells per well on 6-well plates. Cells were treated without or with 0.5 mM succinyl acetone, succinyl acetone+heme (10 µM), 10 µM carbonyl cyanide m-chlorophenyl hydrazone (CCCP), or 10 µM Tin protoporphyrin IX (SnPP). Medium was refreshed every four days. After 10–12 days, cells were fixed in 70% ethanol, stained with 0.5% crystal violet. The images were acquired by using the HP Scanjet 8270.

An in vitro scratch assay was used to examine the effect of heme and heme deficiency on HCC4017 cell migration, as described [29]. Briefly, HCC4017 cells were treated without or with 0.5 mM succinyl acetone, succinyl acetone+heme (10 µM), or 10 µM Tin protoporphyrin IX (SnPP) for 6 days. Then, cells were trypsinized and seeded on culture plates at 95% confluency. The monolayers were scratched with a 200 µl sterile pipette tip and then washed for several times with PBS to remove cell debris. Cells were then maintained in the corresponding media, and migration of the cells was monitored using a microscope. Representative images were captured along the scratch at various time points. Every treatment was performed in triplicates.

## Results

### NSCLC Cells Display Intensified Rates of Oxygen Consumption

We measured and compared the rates of glucose and oxygen consumption in a matched pair of the normal, nonmalignant bronchial epithelial (HBEC30KT) and NSCLC (HCC4017) cells [22], [23]. Table 1 shows that the rates of both glucose and oxygen consumption in HCC4017 cells were elevated, with the elevation of oxygen consumption approaching 2.5-fold (Table 1). However, the ratio of mitochondrial DNA to nuclear DNA, measured as described [30], was not different between the cell lines. These data suggest that while mitochondrial function is not disrupted, mitochondrial respiration is substantially enhanced in NSCLC cells.

**Table 1:** Cancer Cells Substantially Increase the Rates of Glucose and Oxygen Consumption*.

Cell Line	Glu	O_2_	mtDNA
HBEC30KT	10.9±0.6	1.07±0.18	0.63±0.02
HCC4017	15.1±1.0	2.47±0.15	0.61±0.02

* The rates of glucose uptake and oxygen consumption are shown in nmol/min/10^6^ cells, while the mitochondrial DNA level (mtDNA) is shown as the ratio of threshold cycle number of mitochondrial DNA vs. nuclear DNA, measured by real-time PCR.

### The Rate of Heme Biosynthesis and the Expression Levels of Heme Biosynthetic Enzymes are Significantly Increased in Lung Cancer Cells

**Figure 1:**
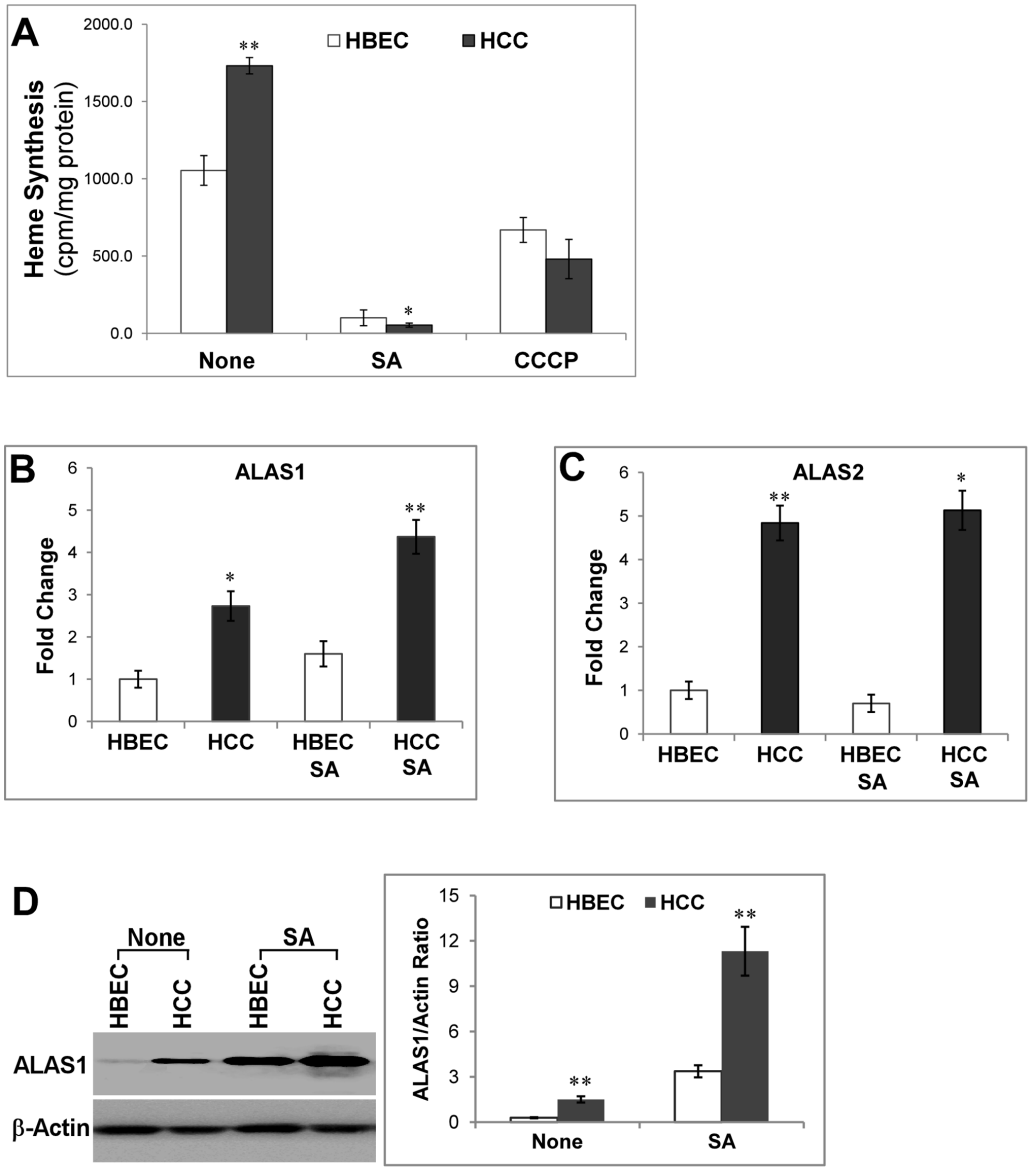
The levels of heme biosynthesis and heme biosynthetic enzymes are enhanced in lung cancer cells. The normal HBEC30KT lung epithelial (HBEC) and NSCLC HCC4017 (HCC) cells were cultured, RNA and proteins were extracted. Transcript levels were detected by using quantitative real-time RT-PCR, and protein levels were detected by using Western blotting. (A) The levels of heme biosynthesis in normal and NSCLC cells. (B) The transcript level of heme biosynthetic enzyme ALAS1 in normal and NSCLC cells. (C) The transcript level of heme biosynthetic enzyme ALAS2 in normal and NSCLC cells. (D) The protein level of heme biosynthetic enzyme ALAS1 in normal and NSCLC cells. The protein level of β-actin in the samples was used for normalization. For statistical analysis, the levels in cancer cells were compared to the levels in normal cells, by using Welch 2-sample t-test. *, p value <0.05; **, p value <0.005.

Heme serves as a prosthetic group in many proteins and enzymes that transport, store and use oxygen and can directly regulate many processes involved in oxygen metabolism [15], [16]. Therefore, we reasoned that the enhanced oxygen consumption in NSCLC cells may be attributable to increased levels of heme and hemoproteins. To test this possibility, we first measured and compared the levels of heme synthesis in NSCLC and normal cells. We found that the rate of heme synthesis was increased significantly in the NSCLC HCC4017 cells, compared to the normal HBEC30KT cells (Figure 1A). The potent inhibitor of heme synthesis, succinyl acetone (SA) [24], [31], inhibited heme synthesis in NSCLC and normal cells, as expected. Succinyl acetone has previously shown to be a specific and potent inhibitor of heme synthesis in diverse cells ranging from HeLa cells, PC12 cells to primary mouse neuronal cells [32]–[37]. Interestingly, the mitochondria uncoupler CCCP (carbonyl cyanide meta-chlorophenylhydrazone) also reduced the rate of heme synthesis, albeit to a lesser extent (Figure 1A). CCCP uncouples oxidative phosphorylation with ATP generation in mitochondria [38]. Because the rate of oxygen consumption is very high, compared to what is needed for heme biosynthesis (Table 1), the amount of oxygen used for heme biosynthesis is negligible [18].

Next, we measured the expression level of ALAS1 (5-aminolevulinic acid synthase 1), which is the rate-limiting enzyme for heme synthesis in nonerythorid cells, including lung cells [39]. We initially measured the ALAS1 transcript levels in NSCLC and normal cells using real time RT-PCR. Figure 1B shows that the transcript level of the nonerythroid ALAS1 gene was indeed increased in the cancer cells. As expected from previous studies [[40], [41] and references therein], inhibition of ALAS activity by succinyl acetone caused induction of ALAS1 in both cancer and normal cells, although the extent of induction appeared to be greater in cancer cells. The erythroid-specific ALAS2 gene is not thought to be expressed in normal lung cells; however, data provided by the Human Protein Atlas (www.proteinatlas.org) suggested that ALAS2 is expressed in 16% of lung cancer tissues. Hence, we also measured the ALAS2 transcript level in lung cells (Figure 1C). Indeed, the ALAS2 transcript level was increased in HCC4017 cells by nearly five-fold. Succinyl acetone did not have a measurable effect on ALAS2 level, as expected, because the expression of ALAS2, unlike ALAS1, is not regulated by heme level [40]. We further confirmed the increase of ALAS protein level in cancer vs. normal cells. Figure 1D shows that ALAS1 protein level was significantly enhanced in the cancer cells, and it was further increased by succinyl acetone. This is consistent with previous studies showing that heme can negatively regulate ALAS1 transcriptionally and posttranscriptionally [40], [41]. We were not able to detect the ALAS2 protein, perhaps due to its low level in lung cells. Although its transcript level is detected in lung cancer cells, its level appears to be significantly lower compared to that of ALAS1.

### The Levels of Heme Uptake Proteins HCP1 and HRG1 and Oxygen-utilizing Hemoproteins are Increased in Lung Cancer Cells

**Figure 2:**
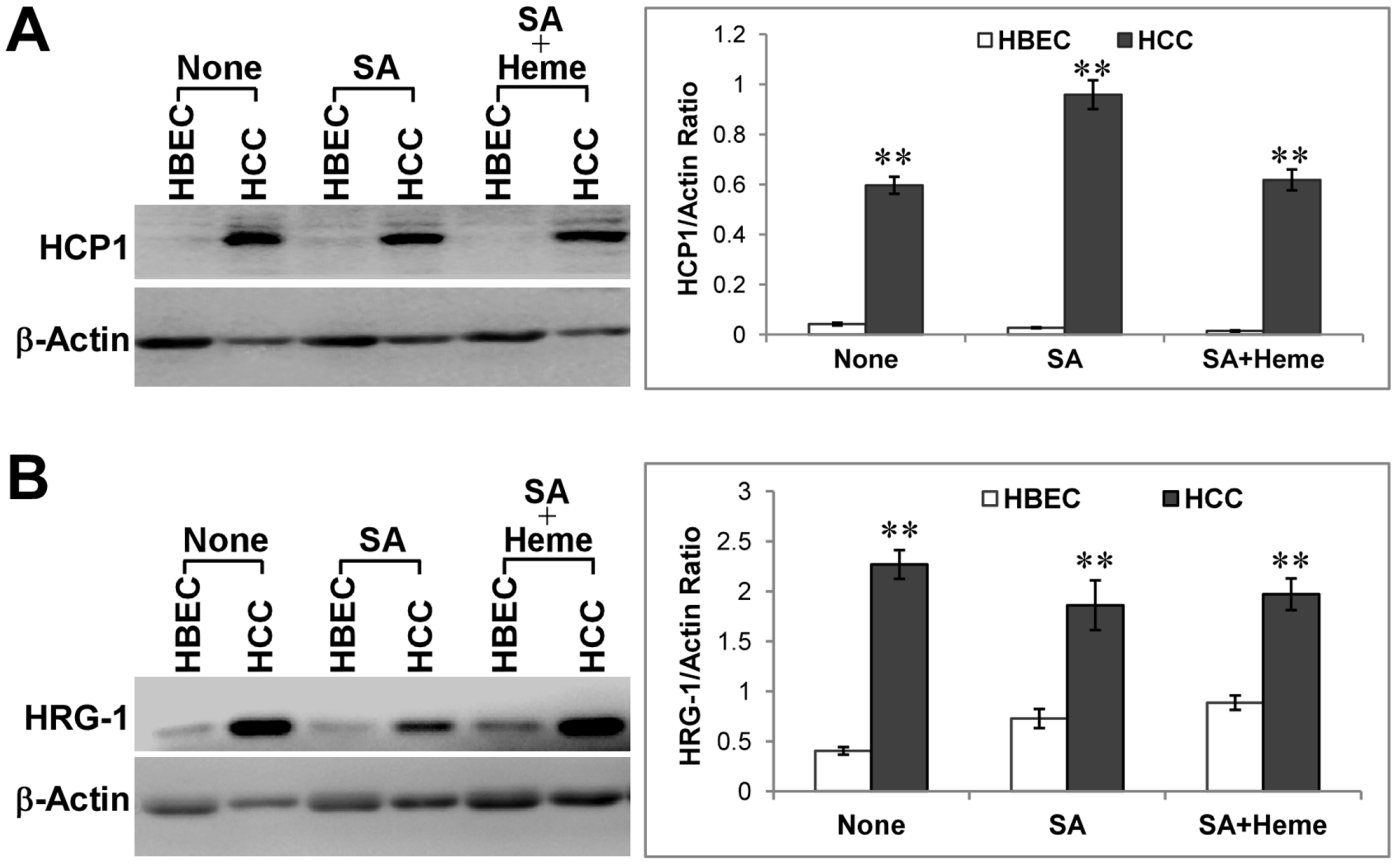
The levels of heme transporters involved in heme uptake are enhanced in lung cancer cells. The normal HBEC30KT lung epithelial (HBEC) and NSCLC HCC4017 (HCC) cells were cultured and treated as indicated. Protein extracts were prepared and the levels of HCP1 and HRG1 were detected by Western blotting. The protein level of β-actin in the samples was used for normalization. (A) The protein level of heme transporter HCP1 in normal and NSCLC cells. (B) The protein level of heme transporter HRG1 in normal and NSCLC cells. For statistical analysis, the levels in cancer cells were compared to the levels in normal cells, by using Welch 2-sample t-test. **, p value <0.005.

To further ascertain the function of heme in NSCLC cells, we compared the levels of two heme transporters HCP1 (heme carrier protein 1) and HRG1 (heme responsive gene 1) [42], [43]. They are expressed and involved in heme uptake in various non-polarized cells [43]–[46]. We found that the levels of HCP1 and HRG1 were dramatically increased in HCC4017 cells, compared to the normal cells (Figure 2A & 2B). Inhibition of heme synthesis by succinyl acetone did not significantly affect HCP1 and HRG1 levels, consistent with previous results in mammalian cells [44].

**Figure 3:**
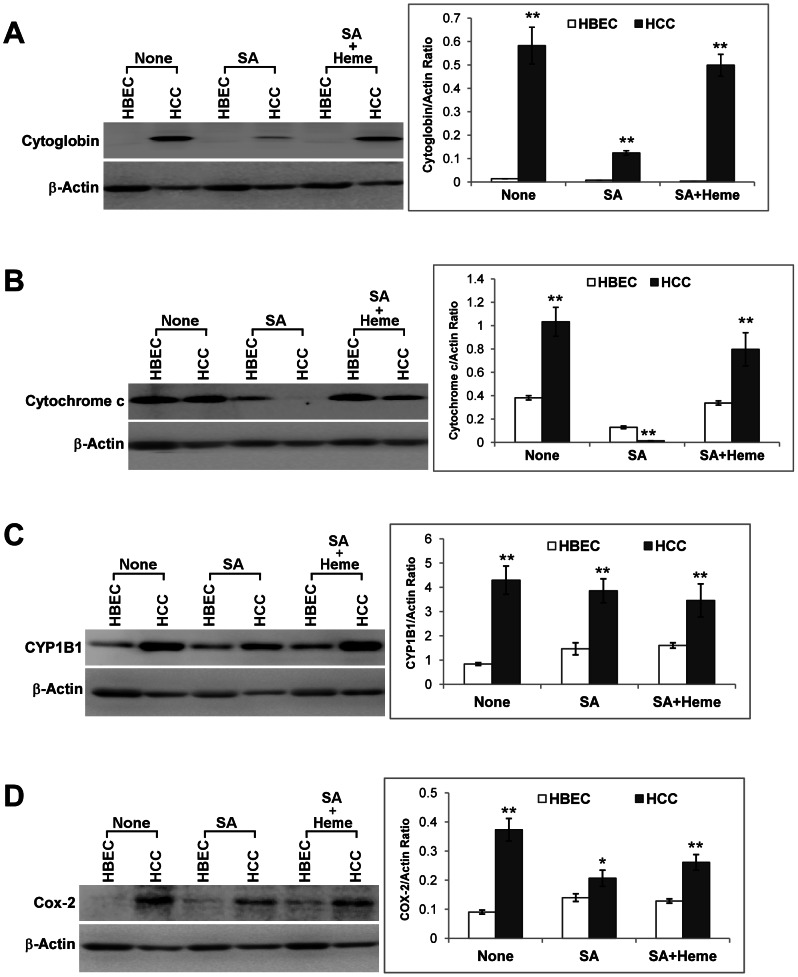
The levels of oxygen utilizing hemoproteins are enhanced in lung cancer cells. The normal HBEC30KT lung epithelial (HBEC) and NSCLC HCC4017 (HCC) cells were cultured and treated as indicated. Protein extracts were prepared and the levels of hemoproteins were detected by Western blotting. The protein level of β-actin was used for normalization. (A) The protein level of cytoglobin in normal and NSCLC cells. (B) The protein level of cytochrome c in normal and NSCLC cells. (C) The protein level of CYP1B1 in normal and NSCLC cells. (D) The protein level of Cox-2 in normal and NSCLC cells. For statistical analysis, the levels in cancer cells were compared to the levels in normal cells, by using Welch 2-sample t-test. *, p value <0.05; **, p value <0.005.

The increase in heme uptake proteins HCP1 and HRG1 can provide additional heme for the production of hemoproteins. We therefore detected the levels of several hemoproteins involved in the transport and utilization of oxygen. Cytoglobin is a hemoprotein expressed in fibroblasts and likely facilitates oxygen transport and utilization [47], [48]. In normal lung epithelial cells, cytoglobin was not detected, but was expressed robustly in HCC4017 cells (Figure 3A). Likewise, the levels of three other hemoproteins involved in oxygen utilization, cytochrome c, CYP1B1 and Cox-2, were also significantly enhanced (Figure 3B–3D). Evidently, while the levels of cytoglobin and cytochrome c were reduced when intracellular heme supply was lowered by culturing cells in heme-depleted medium and by treating with the heme synthesis inhibitor succinyl acetone, affected by heme levels, the levels of CYP1B1 and Cox-2 were not. This may be attributable to the different mechanisms governing the regulation of these proteins. Perhaps heme directly regulates the expression of cytoglobin and cytochrome c, while a heme-independent mechanism contributes to the increased levels of CYP1B1 and Cox-2 in cancer cells. Alternatively, heme may regulate the expression of CYP1B1 and Cox-2, but the heme regulatory concentration may be much lower than that for the expression of cytoglobin and cytochrome c. Hence, the lower heme level in succinyl acetone-treated cells may reduce the levels of cytoglobin and cytochrome c, but not CYP1B1 and Cox-2.

**Figure 4:**
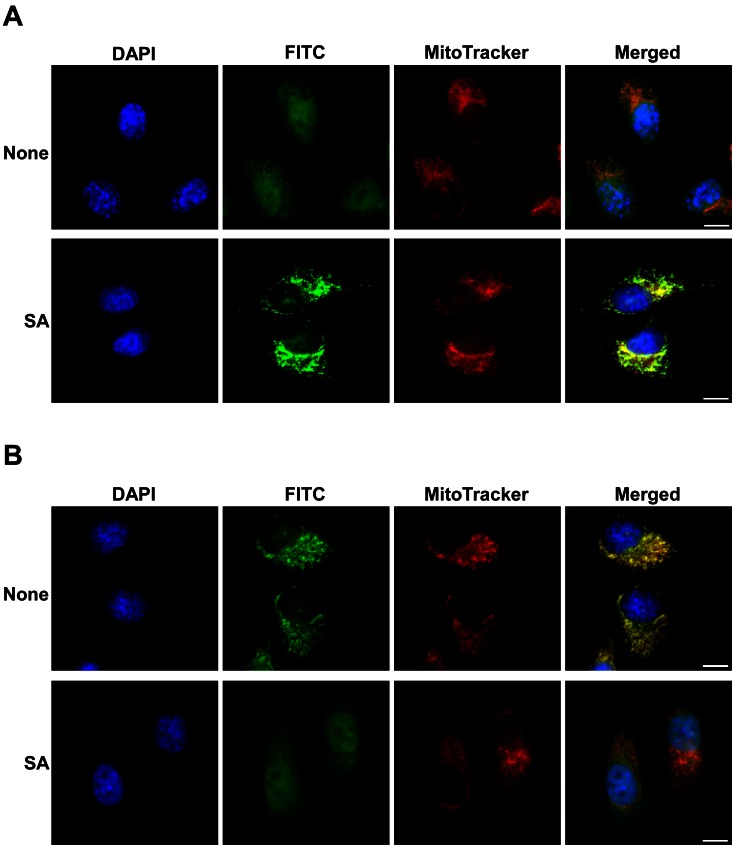
The effect of lowering heme availability on the mitochondrial ALAS1 (A) and cytochrome c (B) proteins. NSCLC HCC4017 (HCC) cells were cultured in regular medium (none) or in heme-depleted medium containing 0.5 mM succinyl acetone (SA). Cells were stained first with anti-ALAS1 or anti-cytochrome c antibodies, and then with FITC-conjugated goat anti-rabbit secondary antibody, MitoTracker, as well as DAPI. FITC, MitoTracker and DAPI fluorescent images were captured and are shown here. The scale bar indicates 10 µm.

The effect of lowering intracellular heme supply on these proteins can also be observed by using immunofluorescence staining. For example, Figure 4A shows that ALAS1 exhibited a mitochondrial localization pattern, particularly when its level was enhanced in cells treated with succinyl acetone. When the background fluorescence was low in panel SA (Figure 4A), FITC fluorescence clearly colocalized with the fluorescence from MitoTracker, and the mitochondrial pattern was much clearer. The pattern in the panel None is less clear, likely because of the background fluorescence caused by lower level of ALAS1 and background antibody staining. Figure 4B shows that cytochrome c exhibited a mitochondrial localization pattern, but its level was reduced in cells treated with succinyl acetone, and its mitochondrial localization was also weakened. The results from indirect immunofluorescence staining are consistent with the results from Western blotting. Together, these results show that enhanced heme synthesis and uptake are associated with increased production of various oxygen-utilizing hemoproteins in cancer cells.

### The Levels of the Heme Biosynthetic Enzyme ALAS, Heme Uptake Proteins HCP1 and HRG1, and Oxygen-utilizing Hemoproteins are Increased in Various Human Tumor Xenografts

**Figure 5:**
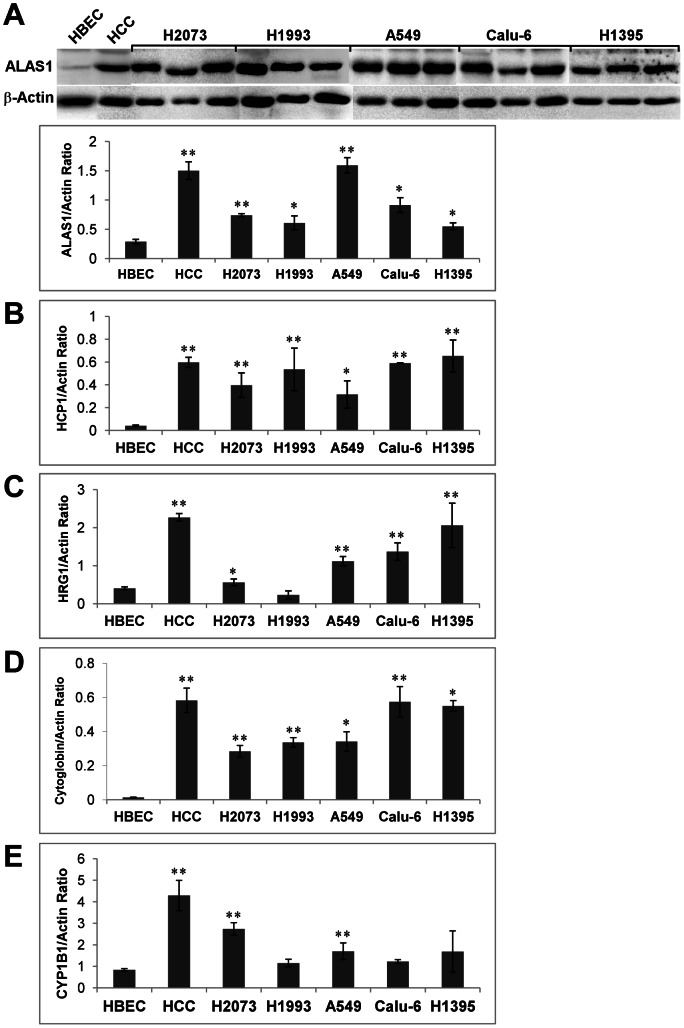
The levels of heme biosynthetic enzymes, heme transporters, and oxygen-utilizing hemoproteins are elevated in various human tumor xenografts. Lysates from the indicated human tumor xenografts were prepared, and the levels of the indicated proteins were detected by Western blotting. The protein level of β-actin was used for normalization. (A) The protein level of ALAS1 in HCC4017 cells and in various human tumor xenografts. (B) The protein level of HCP1 in HCC4017 cells and in various human tumor xenografts. (C) The protein level of HRG1 in HCC4017 cells and in various human tumor xenografts. (D) The protein level of cytoglobin in HCC4017 cells and in various human tumor xenografts. (E) The protein level of CYP1B1 in HCC4017 cells and in various human tumor xenografts. For statistical analysis, the levels in HCC4017 cells and tumors were compared to the levels in normal cells, by using Welch 2-sample t-test. *, p value <0.05; **, p value <0.005.

To determine whether the increase in the rate-limiting heme biosynthetic enzyme ALAS, heme uptake proteins, and various oxygen-utilizing hemoproteins occurs in lung tumors, we evaluated the levels of these proteins in five different human tumor xenografts (Figure 5). Figure 5A shows that in all xenograft tumors, the ALAS1 protein was expressed at levels comparable to that in HCC4017 cells, but significantly higher than that in the normal HBEC30KT cells (see Figure 1D). Likewise, the levels of heme transporters HCP1 (Figure 5B) and HRG1 (Figure 5C) were elevated in the xenograft tumors. The levels of heme-containing, oxygen-binding proteins cytoglobin (Figure 5D) and cytochrome P450 CYP1B1 (Figure 5E) were also enhanced in the tumors. These results show that enhanced heme synthesis, uptake and the synthesis of oxygen-utilizing hemoproteins occur in diverse lung cells and tumors.

### Reducing Heme Availability to NSCLC Cells Preferentially Suppresses Oxygen Consumption

**Figure 6:**
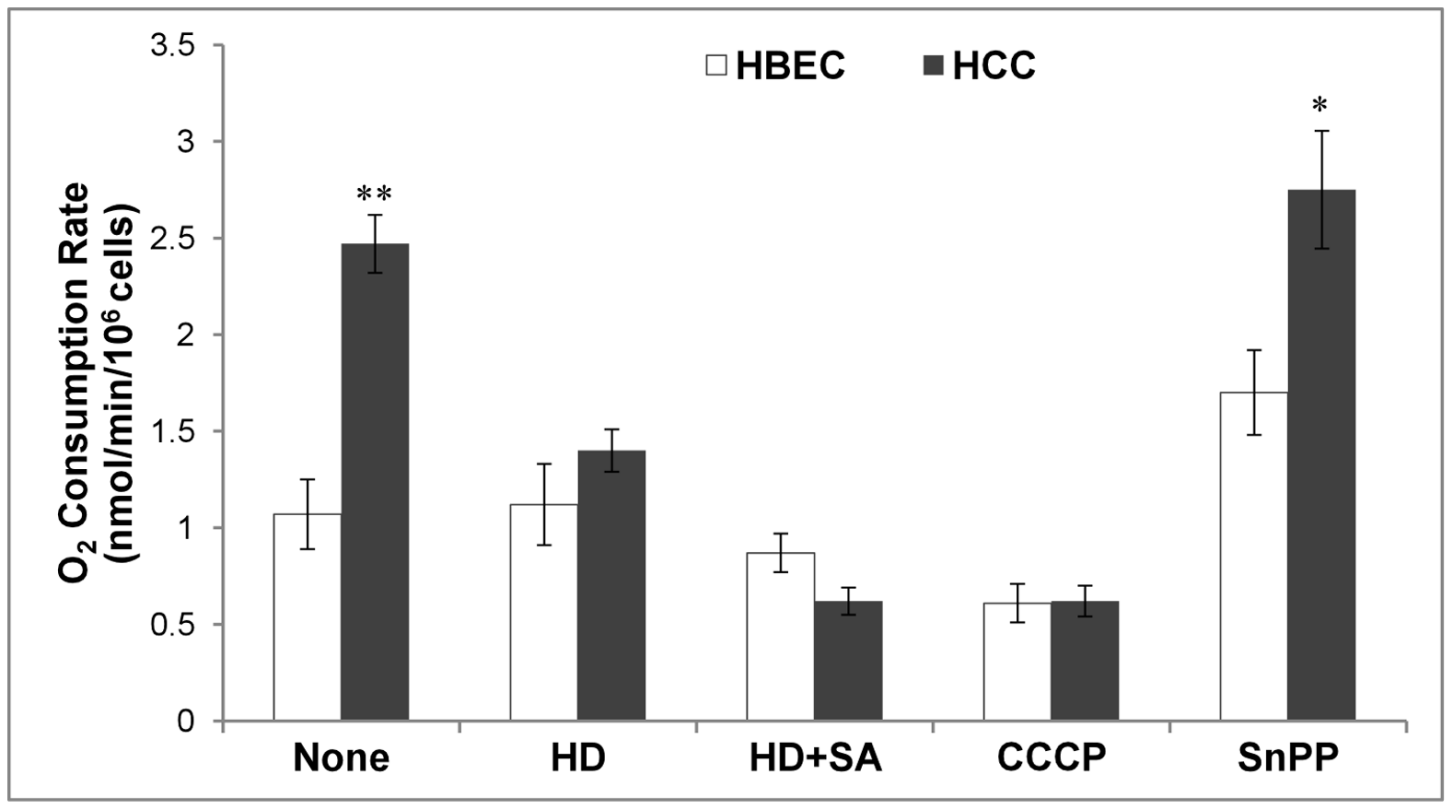
Reducing heme availability suppresses the intensified rate of oxygen consumption in lung cancer cells. The normal HBEC30KT lung epithelial (HBEC) and NSCLC HCC4017 (HCC) cells were cultured and treated in normal medium (None), in medium with heme depleted (HD), in medium with heme depleted and succinyl acetone (HD+SA), and in medium with CCCP. Cells were collected and oxygen consumption rates were detected and plotted here. The values plotted were averages from at least three experiments. For statistical analysis, the levels in cancer cells were compared to the levels in normal cells, by using Welch 2-sample t-test. *, p value <0.05; **, p value <0.005.

Increased synthesis of oxygen-utilizing hemoproteins likely can contribute to intensified oxygen consumption in cancer cells. To test this idea, we examined the effect of depleting heme on the oxygen consumption rate in cancer and normal lung cells. We found that oxygen consumption in the NSCLC cells was selectively reduced when cells were cultured in heme-depleted medium (see Figure 6, HD). In contrast, heme depletion in the medium did not affect oxygen consumption in normal cells. Inhibition of endogenous heme synthesis by succinyl acetone (HD+SA, Figure 6) further reduced oxygen consumption in cancer cells to a level similar to the level in cells treated with the mitochondrial uncoupler CCCP. Succinyl acetone had a lesser effect in the normal cells as well (Figure 6). Evidently, inhibition of heme oxygenase, the enzyme involved in heme degradation, by Tin protoporphyrin (SnPP, Figure 6) did not preferentially affect cancer cells. Notably, these treatments have the same effects on the lung carcinoma A549 cells (not shown). These results show that ample supply of heme is crucial for maintaining oxygen consumption in NSCLC cells at a higher rate than in normal cells. They strongly suggest that enhanced heme synthesis and uptake are required for increased oxygen consumption in NSCLC cells.

### Inhibition of Heme Synthesis and Mitochondrial Function Strongly Suppresses NSCLC Cell Proliferation, Colony Formation and Migration

**Figure 7:**
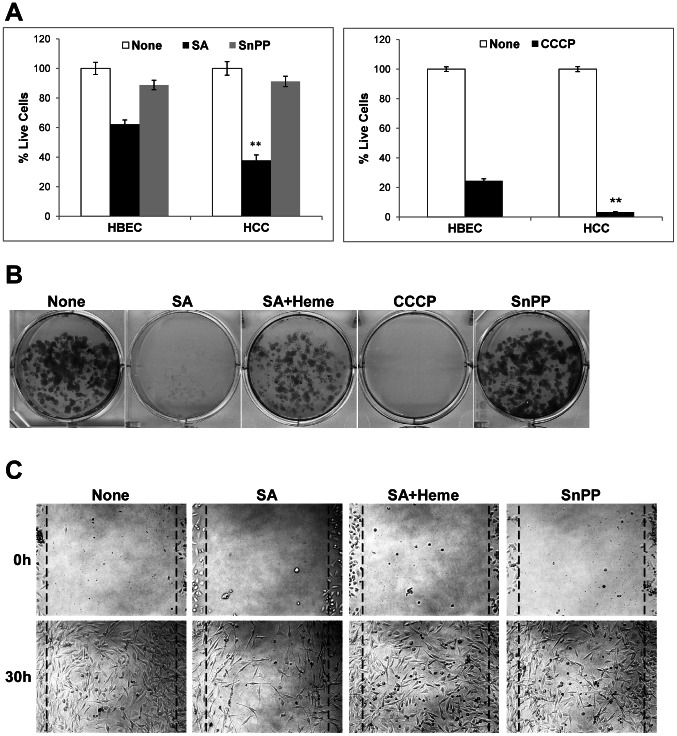
Reducing heme availability suppresses lung cancer cell proliferation, colony formation, and migration. Cells treated with succinyl acetone were also maintained in heme-depleted medium. (A) Reducing heme availability or mitochondrial function preferentially reduces NSCLC cancer cell proliferation. %live cells was calculated by dividing the number of treated cells with the number of untreated cells (seeded with the same number of cells). It shows the relative proliferative rates of treated cells (SA or SnPP) vs. untreated cells (None). (B) Reducing heme availability or mitochondrial function preferentially reduces NSCLC cell colony formation. (C) Reducing heme availability preferentially reduces NSCLC cell migration. For statistical analysis, the levels in cancer cells were compared to the levels in normal cells, by using Welch 2-sample t-test. **, p value <0.005.

To evaluate the effect of inhibiting heme synthesis and mitochondrial function on cancer cell proliferation and function, we examined lung cancer growth rate, colony formation and migration. Figure 7A shows that inhibition of heme synthesis interrupted the growth of the cancer HCC4017 cells more severely than the normal HBEC30KT cells. Likewise, the mitochondrial uncoupler CCCP interrupted HCC4017 cell growth more severely than HBEC30KT cells (Figure 7A). In contrast, inhibition of heme degradation by SnPP did not selectively affect HCC4017 cell proliferation. The same effects were observed when we measured HCC4017 colony formation. As shown in Figure 7B, both succinyl acetone and CCCP completely stopped cancer cell colony formation. Addition of heme largely reversed the effect of inhibition of heme synthesis. As expected, inhibition of heme degradation by Tin protoporphyrin IX (SnPP) did not considerably affect cancer cell colony formation. Further, we examined the effect of inhibiting heme biosynthesis and uptake on HCC4017 cell migration. HCC4017 cell migration was substantially inhibited in medium with heme depleted and with succinyl acetone (Figure 7C). Addition of heme reverses the inhibition on migration. We also attempted to examine the effect of knocking down heme biosynthetic enzymes in the lung cancer cells by using shRNAs that were used to knock down heme biosynthesis in HeLa cells [33]. However, we were not able to obtain clones which exhibit lower levels of heme biosynthesis, likely because the HCC4017 cells have an increased demand for higher levels of heme, lowering heme biosynthesis would diminish their survival. These results show that inhibition of heme availability and function significantly diminishes cancer cell proliferation, colony formation, and migration.

## Discussion

Although many cancer cells exhibit increased glycolysis for energy production in the presence of oxygen [2], [49], emerging experimental data show that mitochondrial respiration is crucial in the bioenergetics of an array of cancer cells [9]. For example, breast cancer cells can generate 80% of their ATP by mitochondrial respiration [50]. Several glioma cell lines were found to be highly dependent on mitochondrial respiration for ATP generation [51]. Likewise, an array of human and mouse cancer cell lines, including HL60, HeLa, 143B and U937, utilize oxygen metabolism and respiration to support their growth [52]. Even under hypoxia, mitochondrial respiration can generate about 40% of the cellular energy in cancer cells [14]. In this report, by using a pair of cell lines representing normal, nonmalignant HBEC and NSCLC cells developed from the same patient, we directly compared the rates of oxygen consumption in normal and cancer cells. Our data show that oxygen consumption and mitochondrial respiration are intensified in cancer cells. This strengthens the idea that mitochondrial respiration can be crucial for cancer cell bioenergetics.

Importantly, our data show that cancer cells exhibit elevated heme synthesis, uptake, and incorporation into oxygen-utilizing hemoproteins. The increased heme flux and the synthesis of hemoproteins are likely required for the intensified oxygen consumption, because limiting heme synthesis and uptake reduced oxygen consumption to the same level as inhibition of mitochondrial function (Figure 6). Our data also show that the levels of rate-limiting heme biosynthetic enzyme ALAS and the heme transporters HCP1 and HRG1, which are likely involved in heme uptake [43]–[46], were strongly increased in NSCLC cells (Figures 1 and 2). Further, we show that several representative hemoproteins involved in oxygen transport and utilization were upregulated in NSCLC cells (Figures 3 and 5). These include cytoglobin [47], [48], which may transport oxygen; cytochrome c, which represents hemoproteins in the mitochondrial respiratory chain complexes; CYP1B1, which is a cytochrome P450 enzyme; and Cox-2, a cyclooxygenase. Heme is known to stimulate the synthesis of many hemoproteins [15], [53], although to varying degrees, as shown in Figure 3. The increased heme synthesis and uptake in cancer cells would presumably increase the synthesis of hemoproteins, as we show here.

It is logically expected that the increased levels of oxygen-utilizing hemoproteins would cause intensified oxygen consumption in cancer cells. Conversely, inhibition of heme synthesis and uptake would suppress oxygen consumption. The suppression of oxygen consumption and mitochondrial respiration would result in diminished cellular energy supply, inhibiting cancer cell proliferation, migration and colony formation. This line of reasoning is supported by the data showing that oxygen consumption is increased in cancer cells and is suppressed to the level similar or lower than in normal cells by reducing heme uptake and synthesis (Figure 6). Further, our data also show that reducing intracellular heme also inhibited cell proliferation, colony formation and cell migration (Figure 7). Although heme synthesis requires oxygen, the intracellular level of heme and heme synthesis is very low (less than 0.1 micromolar) [54], [55] compared to oxygen consumption (see also Table 1). Hence, the enhanced synthesis of heme per se cannot account for any increase in oxygen consumption in NSCLC cells. In summary, our data show that enhanced heme synthesis and uptake in NSCLC cells lead to enhanced levels of oxygen-utilizing hemoproteins, which causes enhanced oxygen consumption, cellular energy production and biosynthesis, thereby driving cancer cell proliferation, migration and colony formation. Additionally, we found that inhibition of heme oxygenase did not selectively affect oxygen consumption, proliferation or colony formation by NSCLC cells (Figures 6 and 7). Although a previous study [56] showed that heme oxygenase is involved in the development of hereditary leiomyomatosis and renal-cell cancer (HLRCC), our data here show that it does not play an important role in lung cancer. Hence, it is likely that for NSCLC cell progression, a higher heme level per se and enhanced heme function, not heme degradation products, are key promoting factors.

The increase in heme biosynthetic and uptake proteins and other oxygen-utilizing hemoproteins is observed in an array of human tumor xenografts (Figure 4), with different phenotypes and drug sensitivities. This shows that the increase in heme and hemoproteins is a general feature of lung cancer cells. This can have important implications in the treatment of lung cancers. Interestingly, the Km of heme biosynthetic enzymes and mitochondria for oxygen is very low (less than 1 µM, about 0.1% oxygen saturation) [18], [57], [58]. This is below the oxygen levels experienced by human cells under hypoxia. Thus, both heme synthesis and mitochondrial respiration can be maintained at significant levels under hypoxia. Indeed, this is what was observed in cancer cells [14]. Very likely, the enhanced expression of hemoproteins in cancer cells further promotes the binding and metabolizing of oxygen by various enzymes. Our data show that enhanced heme function and oxygen consumption are likely an important mechanism contributing to the progression of lung cancer cells. This mechanism may not limit to only NSCLC cells. Different cancer cells have acquired different ways to obtain cellular energy. For example, glioma cells are highly dependent on glycolysis, while breast cancer cells generate up to 80% of ATP from oxidative phosphorylation/mitochondrial respiration [9], [50], [51]. Enhanced heme function and oxygen consumption are likely a mechanism contributing to the progression of these breast cancer cells. For such cancer cells, heme can provide a potential target applicable to both hypoxic and nonhypoxic regions of tumors.

Targeting heme function can diminish mitochondrial respiration and energy generation, as well as other cellular processes controlling cancer cell function. Experimental studies in the past decade have demonstrated that heme can directly bind to and control the activities of a wide array of cellular regulators, such as the transcriptional factor Bach1 [59], the heme-regulated eIF2α kinase [60], [61], the ras-ERK signaling pathway [24], and the essential miRNA processing factor DGCR8 [62]. Diminishing intracellular heme levels therefore can suppress both energy generation and other cellular processes crucial for cancer cell progression. Thus, targeting heme function can provide an effective way to combat lung cancers, for which the currently available chemotherapeutic agents are mainly palliative [63], [64].

### Conclusions

**Figure 8:**
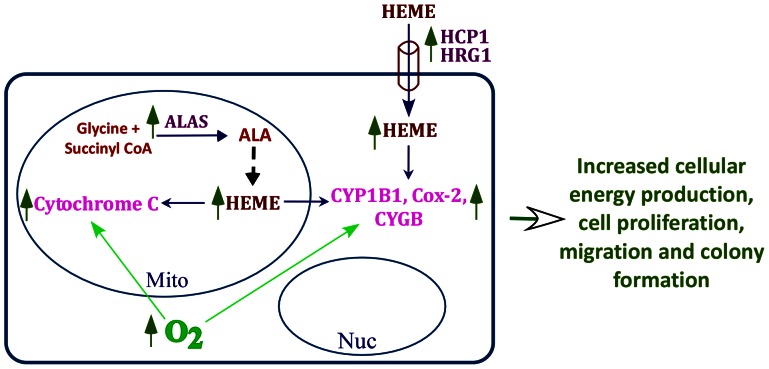
Cartoon illustrating key bioenergetics changes in NSCLC cells. Cancer cells exhibit enhanced expression levels of the rate-limiting heme biosynthetic enzyme, 5-aminolevulinic acid synthase (ALAS) and the heme uptake proteins HCP1 and HRG1. As such, heme availability in cancer cells is substantially increased, leading to increased production and levels of various oxygen-utilizing hemoproteins, such as cytochrome c, cytoglobin, Cox-2 and cytochrome P450. The increase in these hemoproteins ultimately leads to intensified oxygen consumption and the generation of cellular energy by respiration. This in turn causes increased cellular energy production, cell proliferation, migration and colony formation.

Our comparative study of normal and NSCLC cells and analysis of human xenograft tumors uncovers a key bioenergetic alteration in lung cancer cells. Evidently, in cancer cells, the levels of the rate-limiting heme biosynthetic enzyme ALAS and heme uptake proteins HCP1 and HRG1 are strongly enhanced (Figure 8). Increasing the availability of a key component, heme, can lead to enhanced levels of an array of oxygen-utilizing hemoproteins in mitochondria, as well as in other cellular compartments (Figure 8). The increase in these proteins and enzymes enable cancer cells to intensify mitochondrial respiration and oxygen consumption, thereby generating ample cellular energy to fuel cancer cell progression. Suppressing heme availability, like inhibiting mitochondrial respiration, arrest cancer cell progression. These results suggest that heme function and mitochondrial respiration are key factors in lung cancer cell progression. They provide a basis for further studies to understand bioenergetics in advanced cancer cells and to design novel cancer therapeutics by targeting heme and mitochondrial function.

## References

[1] Minami S (1923) Respiration and glycolysis. Biochem. Z. 142: 94–113.

[2] Warburg O (1930) The Metabolism of Tumours. London: Constable & Co.

[3] Vander Heiden MG, Locasale JW, Swanson KD, Sharfi H, Heffron GJ, et al (2010) Evidence for an alternative glycolytic pathway in rapidly proliferating cells. Science 329: 1492–9. 20847263 10.1126/science.1188015 PMC3030121

[4] Hitosugi T, Kang S, Vander Heiden MG, Chung TW, Elf S, et al (2009) Tyrosine phosphorylation inhibits PKM2 to promote the Warburg effect and tumor growth. Sci Signal 2: ra73. 19920251 10.1126/scisignal.2000431 PMC2812789

[5] Anastasiou D, Poulogiannis G, Asara JM, Boxer MB, Jiang JK, et al (2011) Inhibition of pyruvate kinase M2 by reactive oxygen species contributes to cellular antioxidant responses. Science 334: 1278–83. 22052977 10.1126/science.1211485 PMC3471535

[6] Eng C, Kiuru M, Fernandez MJ, Aaltonen LA (2003) A role for mitochondrial enzymes in inherited neoplasia and beyond. Nat Rev Cancer 3: 193–202. 12612654 10.1038/nrc1013

[7] DeBerardinis RJ, Thompson CB (2012) Cellular metabolism and disease: what do metabolic outliers teach us? Cell 148: 1132–44. 22424225 10.1016/j.cell.2012.02.032 PMC3337773

[8] Birsoy K, Sabatini DM, Possemato R (2012) Untuning the tumor metabolic machine: Targeting cancer metabolism: a bedside lesson. Nat Med 18: 1022–3. 22772555 10.1038/nm.2870

[9] Jose C, Bellance N, Rossignol R (2011) Choosing between glycolysis and oxidative phosphorylation: a tumor’s dilemma? Biochim Biophys Acta 1807: 552–61. 20955683 10.1016/j.bbabio.2010.10.012

[10] Bonuccelli G, Tsirigos A, Whitaker-Menezes D, Pavlides S, Pestell RG, et al (2010) Ketones and lactate “fuel” tumor growth and metastasis: Evidence that epithelial cancer cells use oxidative mitochondrial metabolism. Cell Cycle 9: 3506–14. 20818174 10.4161/cc.9.17.12731 PMC3047616

[11] Moreno-Sanchez R, Rodriguez-Enriquez S, Marin-Hernandez A, Saavedra E (2007) Energy metabolism in tumor cells. Febs J 274: 1393–418. 17302740 10.1111/j.1742-4658.2007.05686.x

[12] Whitaker-Menezes D, Martinez-Outschoorn UE, Flomenberg N, Birbe RC, Witkiewicz AK, et al (2011) Hyperactivation of oxidative mitochondrial metabolism in epithelial cancer cells in situ: visualizing the therapeutic effects of metformin in tumor tissue. Cell Cycle 10: 4047–64. 22134189 10.4161/cc.10.23.18151 PMC3272287

[13] Kaambre T, Chekulayev V, Shevchuk I, Karu-Varikmaa M, Timohhina N, et al (2012) Metabolic control analysis of cellular respiration in situ in intraoperational samples of human breast cancer. J Bioenerg Biomembr 44: 539–58. 22836527 10.1007/s10863-012-9457-9

[14] Frezza C, Zheng L, Tennant DA, Papkovsky DB, Hedley BA, et al (2011) Metabolic profiling of hypoxic cells revealed a catabolic signature required for cell survival. PLoS One 6: e24411. 21912692 10.1371/journal.pone.0024411 PMC3166325

[15] Padmanaban G, Venkateswar V, Rangarajan PN (1989) Haem as a multifunctional regulator. Trends Biochem Sci 14: 492–6. 2696180 10.1016/0968-0004(89)90182-5

[16] Mense SM, Zhang L (2006) Heme: a versatile signaling molecule controlling the activities of diverse regulators ranging from transcription factors to MAP kinases. Cell Res 16: 681–92. 16894358 10.1038/sj.cr.7310086

[17] Ortiz de Montellano PR (2009) Hemes in Biology. In: Wiley Encyclopedia of Chemical Biology, editors. West Sussex: John Wiley & Sons, Ltd. 240–9.

[18] Andrew TL, Riley PG, Dailey HA (1990) Regulation of heme biosynthesis in higher animals. In: Biosynthesis of heme and cholorophylls, HA Dailey, editors. New York: Green Pub. Associates and Wiley-Interscience. 183–232.

[19] Jemal A, Siegel R, Xu J, Ward E (2010) Cancer statistics, 2010. CA Cancer J Clin 60: 277–300. 20610543 10.3322/caac.20073

[20] Organization WH (2011) Cancer. Fact sheet no. 297. World Health Organization.

[21] Mountain CF (2000) The international system for staging lung cancer. Semin Surg Oncol 18: 106–15. 10657912 10.1002/(sici)1098-2388(200003)18:2<106::aid-ssu4>3.0.co;2-p

[22] Ramirez RD, Sheridan S, Girard L, Sato M, Kim Y, et al (2004) Immortalization of human bronchial epithelial cells in the absence of viral oncoproteins. Cancer Res 64: 9027–34. 15604268 10.1158/0008-5472.CAN-04-3703

[23] Whitehurst AW, Bodemann BO, Cardenas J, Ferguson D, Girard L, et al (2007) Synthetic lethal screen identification of chemosensitizer loci in cancer cells. Nature 446: 815–9. 17429401 10.1038/nature05697

[24] Zhu Y, Hon T, Ye W, Zhang L (2002) Heme deficiency interferes with the Ras-mitogen-activated protein kinase signaling pathway and expression of a subset of neuronal genes. Cell Growth Differ 13: 431–9. 12354752

[25] Papandreou I, Cairns RA, Fontana L, Lim AL, Denko NC (2006) HIF-1 mediates adaptation to hypoxia by actively downregulating mitochondrial oxygen consumption. Cell Metab 3: 187–97. 16517406 10.1016/j.cmet.2006.01.012

[26] Shedlofsky SI, Sinclair PR, Bonkovsky HL, Healey JF, Swim AT, et al (1987) Haem synthesis from exogenous 5-aminolaevulinate in cultured chick-embryo hepatocytes. Effects of inducers of cytochromes P-450. Biochem J 248: 229–36. 3435440 10.1042/bj2480229 PMC1148523

[27] Pejznochova M, Tesarova M, Hansikova H, Magner M, Honzik T, et al (2010) Mitochondrial DNA content and expression of genes involved in mtDNA transcription, regulation and maintenance during human fetal development. Mitochondrion 10: 321–9. 20096380 10.1016/j.mito.2010.01.006

[28] Sullivan LA, Carbon JG, Roland CL, Toombs JE, Nyquist-Andersen M, et al (2010) r84, a novel therapeutic antibody against mouse and human VEGF with potent anti-tumor activity and limited toxicity induction. PLoS One 5: e12031. 20700512 10.1371/journal.pone.0012031 PMC2917360

[29] Liang CC, Park AY, Guan JL (2007) In vitro scratch assay: a convenient and inexpensive method for analysis of cell migration in vitro. Nat Protoc 2: 329–33. 17406593 10.1038/nprot.2007.30

[30] Pejznochova M, Tesarova M, Honzik T, Hansikova H, Magner M, et al (2008) The developmental changes in mitochondrial DNA content per cell in human cord blood leukocytes during gestation. Physiol Res 57: 947–55. 18052680 10.33549/physiolres.931246

[31] De Matteis F, Marks GS (1983) The effect of N-methylprotoporphyrin and succinyl-acetone on the regulation of heme biosynthesis in chicken hepatocytes in culture. FEBS Lett 159: 127–31. 6688226 10.1016/0014-5793(83)80430-x

[32] Ye W, Zhang L (2004) Heme controls the expression of cell cycle regulators and cell growth in HeLa cells. Biochem Biophys Res Commun 315: 546–54. 14975735 10.1016/j.bbrc.2004.01.092

[33] Yao X, Balamurugan P, Arvey A, Leslie C, Zhang L (2010) Heme controls the regulation of protein tyrosine kinases Jak2 and Src. Biochem Biophys Res Commun 403: 30–5. 21036157 10.1016/j.bbrc.2010.10.101 PMC3038409

[34] Sengupta A, Hon T, Zhang L (2005) Heme deficiency suppresses the expression of key neuronal genes and causes neuronal cell death. Brain Res Mol Brain Res 137: 23–30. 15950757 10.1016/j.molbrainres.2005.02.007

[35] Chernova T, Nicotera P, Smith AG (2006) Heme deficiency is associated with senescence and causes suppression of N-methyl-D-aspartate receptor subunits expression in primary cortical neurons. Mol Pharmacol 69: 697–705. 16306232 10.1124/mol.105.016675

[36] Chernova T, Steinert JR, Guerin CJ, Nicotera P, Forsythe ID, et al (2007) Neurite degeneration induced by heme deficiency mediated via inhibition of NMDA receptor-dependent extracellular signal-regulated kinase 1/2 activation. J Neurosci 27: 8475–85. 17687025 10.1523/JNEUROSCI.0792-07.2007 PMC6672932

[37] Yin L, Wu N, Curtin JC, Qatanani M, Szwergold NR, et al (2007) Rev-erbalpha, a heme sensor that coordinates metabolic and circadian pathways. Science 318: 1786–9. 18006707 10.1126/science.1150179

[38] Wallace KB, Starkov AA (2000) Mitochondrial targets of drug toxicity. Annu Rev Pharmacol Toxicol 40: 353–88. 10836141 10.1146/annurev.pharmtox.40.1.353

[39] Anderson KE, Sassa S, Bishop DF, Desnick RJ (2009) Disorders of heme biosynthesis: X-linked sideroblastic anemia and the porphyrias. In: Chapter The metabolic, molecular bases of inherited disease, CR Scriver, AL Beaudt, WS Sly, D Valle, C Barton, KW Kinzler, B Vogelstein, editors. New York: The McGraw-Hill Companies, Inc. pp. 124: 1–53.

[40] Anderson KE, Sassa S, Bishop DF, Desnick RJ (2001) Disorders of heme biosynthesis: X-linked sideroblastic anemia and the porphyrias. In: The metabolic and molecular bases of inherited disease, CR Scriver, AL Beaudt, WS Sly, D Valle, C Barton, KW Kinzler, B Vogelstein, editors. New York: The McGraw-Hill Companies, Inc. 2991–3062.

[41] Zhang L, Sessoms R (2011) Heme biosynthesis and degradation: What happens when it goes haywire?. In: HEME BIOLOGY: The Secret Life of Heme in Regulating Diverse Biological Processes, L Zhang, editors. Singapore: World Scientific Publishing Company p.

[42] Latunde-Dada GO, Takeuchi K, Simpson RJ, McKie AT (2006) Haem carrier protein 1 (HCP1): Expression and functional studies in cultured cells. FEBS Lett 580: 6865–70. 17156779 10.1016/j.febslet.2006.11.048

[43] Rajagopal A, Rao AU, Amigo J, Tian M, Upadhyay SK, et al (2008) Haem homeostasis is regulated by the conserved and concerted functions of HRG-1 proteins. Nature 453: 1127–31. 18418376 10.1038/nature06934 PMC4058867

[44] Khan AA, Quigley JG (2011) Control of intracellular heme levels: heme transporters and heme oxygenases. Biochim Biophys Acta 1813: 668–82. 21238504 10.1016/j.bbamcr.2011.01.008 PMC3079059

[45] Dang TN, Bishop GM, Dringen R, Robinson SR (2009) The putative heme transporter HCP1 is expressed in cultured astrocytes and contributes to the uptake of hemin. Glia 58: 55–65. 10.1002/glia.20901 19533605

[46] Shayeghi M, Latunde-Dada GO, Oakhill JS, Laftah AH, Takeuchi K, et al (2005) Identification of an intestinal heme transporter. Cell 122: 789–801. 16143108 10.1016/j.cell.2005.06.025

[47] Burmester T, Ebner B, Weich B, Hankeln T (2002) Cytoglobin: a novel globin type ubiquitously expressed in vertebrate tissues. Mol Biol Evol 19: 416–21. 11919282 10.1093/oxfordjournals.molbev.a004096

[48] Trent JT 3rd, Hargrove MS (2002) A ubiquitously expressed human hexacoordinate hemoglobin. J Biol Chem 277: 19538–45. 11893755 10.1074/jbc.M201934200

[49] Dang CV (2009) PKM2 tyrosine phosphorylation and glutamine metabolism signal a different view of the Warburg effect. Sci Signal 2: pe75. 19920249 10.1126/scisignal.297pe75

[50] Guppy M, Leedman P, Zu X, Russell V (2002) Contribution by different fuels and metabolic pathways to the total ATP turnover of proliferating MCF-7 breast cancer cells. Biochem J 364: 309–15. 11988105 10.1042/bj3640309 PMC1222574

[51] Griguer CE, Oliva CR, Gillespie GY (2005) Glucose metabolism heterogeneity in human and mouse malignant glioma cell lines. J Neurooncol 74: 123–33. 16193382 10.1007/s11060-004-6404-6

[52] Herst PM, Berridge MV (2007) Cell surface oxygen consumption: a major contributor to cellular oxygen consumption in glycolytic cancer cell lines. Biochim Biophys Acta 1767: 170–7. 17266920 10.1016/j.bbabio.2006.11.018

[53] Zhang L (2011) HEME BIOLOGY: The Secret Life of Heme in Regulating Diverse Biological Processes. Singapore: World Scientific Publishing Company.

[54] Ryter SW, Tyrrell RM (2000) The heme synthesis and degradation pathways: role in oxidant sensitivity. Heme oxygenase has both pro- and antioxidant properties. Free Radic Biol Med 28: 289–309. 11281297 10.1016/s0891-5849(99)00223-3

[55] Sassa S (2004) Why heme needs to be degraded to iron, biliverdin IXalpha, and carbon monoxide? Antioxid Redox Signal 6: 819–24. 15345141 10.1089/ars.2004.6.819

[56] Frezza C, Zheng L, Folger O, Rajagopalan KN, MacKenzie ED, et al (2011) Haem oxygenase is synthetically lethal with the tumour suppressor fumarate hydratase. Nature 477: 225–8. 21849978 10.1038/nature10363

[57] Chance B (1965) Reaction of oxygen with the respiratory chain in cells and tissues. J Gen Physiol 49 Suppl: 163–95 10.1085/jgp.49.1.163 PMC2195456 4285727

[58] Labbe-Bois R, Labbe P (1990) Tetrapyrrole and heme biosynthesis in the yeast Sacchromyces cerevisiae. In: Biosynthesis of heme and cholorophylls, HA Dailey, editors. New York: Green Pub. Associates and Wiley-Interscience. 235–85.

[59] Ogawa K, Sun J, Taketani S, Nakajima O, Nishitani C, et al (2001) Heme mediates derepression of Maf recognition element through direct binding to transcription repressor Bach1. Embo J 20: 2835–43. 11387216 10.1093/emboj/20.11.2835 PMC125477

[60] Chen JJ, London IM (1995) Regulation of protein synthesis by heme-regulated eIF-2 alpha kinase. Trends Biochem Sci 20: 105–8. 7709427 10.1016/s0968-0004(00)88975-6

[61] de Haro C, Mendez R, Santoyo J (1996) The eIF-2alpha kinases and the control of protein synthesis. Faseb J 10: 1378–87. 8903508 10.1096/fasebj.10.12.8903508

[62] Faller M, Matsunaga M, Yin S, Loo JA, Guo F (2007) Heme is involved in microRNA processing. Nat Struct Mol Biol 14: 23–9. 17159994 10.1038/nsmb1182

[63] Escobar M, Velez M, Belalcazar A, Santos ES, Raez LE (2011) The role of proteasome inhibition in nonsmall cell lung cancer. J Biomed Biotechnol 2011: 806506. 21629760 10.1155/2011/806506 PMC3100637

[64] Sangodkar J, Katz S, Melville H, Narla G (2010) Lung adenocarcinoma: lessons in translation from bench to bedside. Mt Sinai J Med 77: 597–605. 21105123 10.1002/msj.20226

